# Hemorrhagic hepatocellular carcinoma managed with urgent transarterial particle embolization: A case report

**DOI:** 10.1016/j.radcr.2026.07.087

**Published:** 2026-08-22

**Authors:** Obinna Agwuncha, Shivam Khatri, Matthew Smith, Nii Koney

**Affiliations:** a Department of Interventional Radiology, Jamaica Hospital Medical Center, New York, NY, USA; b Department of Radiology, Jacobi Medical Center, Bronx, NY, USA; c CUNY School of Medicine, City University of New York, New York, NY, USA; d Department of Interventional Radiology, One Brooklyn Health/Brookdale Hospital Medical Center, Brooklyn, NY, USA

**Keywords:** Hepatocellular carcinoma, Hemorrhagic liver tumor, Transarterial embolization, Interventional radiology, Case report

## Abstract

Hepatocellular carcinoma (HCC) is the most common primary malignancy of the liver and a major cause of cancer-related mortality worldwide. Although many cases follow an indolent course, spontaneous hemorrhage from HCC is a rare but life-threatening complication that requires rapid diagnosis and intervention. A 77-year-old man with hypertension, type 2 diabetes mellitus, and chronic kidney disease presented with chest pain and a new-onset generalized seizure. During hospitalization, he developed severe acute blood loss anemia, with hemoglobin declining to approximately 5.7-5.8 g/dL. Cross-sectional imaging revealed a large hypervascular left hepatic lobe mass with intratumoral hemorrhage and hemoperitoneum, suspicious for hepatocellular carcinoma. Although he remained hemodynamically stable, the lesion was considered at high risk for recurrent or catastrophic bleeding. Because transfer to a tertiary hepatobiliary center was initially declined, he underwent inpatient ultrasound-guided biopsy followed by urgent transarterial particle embolization of the left hepatic artery. Embolization achieved complete angiographic stasis without complications. Follow-up MRI demonstrated expected post-embolization necrotic evolution of the treated lesion. This case highlights that preserved hemodynamics do not eliminate the need for urgent hemorrhage control when a large hypervascular hepatic tumor carries a substantial risk of rebleeding.

## Introduction

Hepatocellular carcinoma (HCC) accounts for approximately 75%-85% of primary liver malignancies and remains a major global health burden [1]. Common risk factors include chronic viral hepatitis, alcohol-related liver disease, and metabolic dysfunction-associated fatty liver disease [1,2]. While many patients are diagnosed through surveillance or present with gradually progressive symptoms, spontaneous rupture or hemorrhage of HCC is an uncommon but highly lethal complication, occurring in approximately 3%-15% of cases with mortality rates ranging from 25% to 75% without prompt intervention [[3], [4], [5]]. For patients presenting with acute hemorrhage or those who are not surgical candidates, transarterial embolization (TAE) offers a minimally invasive option to achieve hemostasis, reduce tumor vascularity, and prevent recurrent bleeding [[6], [7], [8]]. We report a case of hemorrhagic hepatocellular carcinoma presenting with severe acute blood loss anemia but preserved hemodynamics, successfully managed with urgent transarterial particle embolization because of the high risk of rebleeding.

## Case presentation

A 77-year-old man with a medical history of hypertension, type 2 diabetes mellitus, and chronic kidney disease presented to the emergency department on July 10, 2025, with midsternal chest pain rated 5/10 that had gradually improved. Upon arrival, he experienced a witnessed generalized tonic–clonic seizure lasting approximately 2 minutes, followed by brief postictal confusion and subsequent return to baseline mental status. He denied trauma and had no previously known history of chronic liver disease.

Initial laboratory evaluation demonstrated anemia, with a hemoglobin level of 8.8 g/dL, as well as hyperglycemia, acute kidney injury, mild hyponatremia, nonanion gap metabolic acidosis, lactic acidosis, and mild transaminitis. Admission liver chemistry testing showed an aspartate aminotransferase level of 70 U/L, alanine aminotransferase level of 50 U/L, alkaline phosphatase level of 74 U/L, total bilirubin level of 0.5 mg/dL, albumin level of 2.5 g/dL, and total protein level of 5.0 g/dL.

Electrocardiography demonstrated normal sinus rhythm with a right bundle branch block. A mild troponin elevation was attributed to acute anemia. Computed tomography of the head was unremarkable. The seizure was considered multifactorial in the setting of metabolic derangements and anemia, and levetiracetam was initiated.

Serial laboratory testing demonstrated worsening severe anemia, with the hemoglobin level declining from 8.8 g/dL at presentation to approximately 5.7-5.8 g/dL during hospitalization. No administration of packed red blood cells was identified in the records available for review. Despite the severity of the anemia, the patient remained hemodynamically stable, without documented sustained hypotension or vasopressor requirement.

**Fig. 1:**
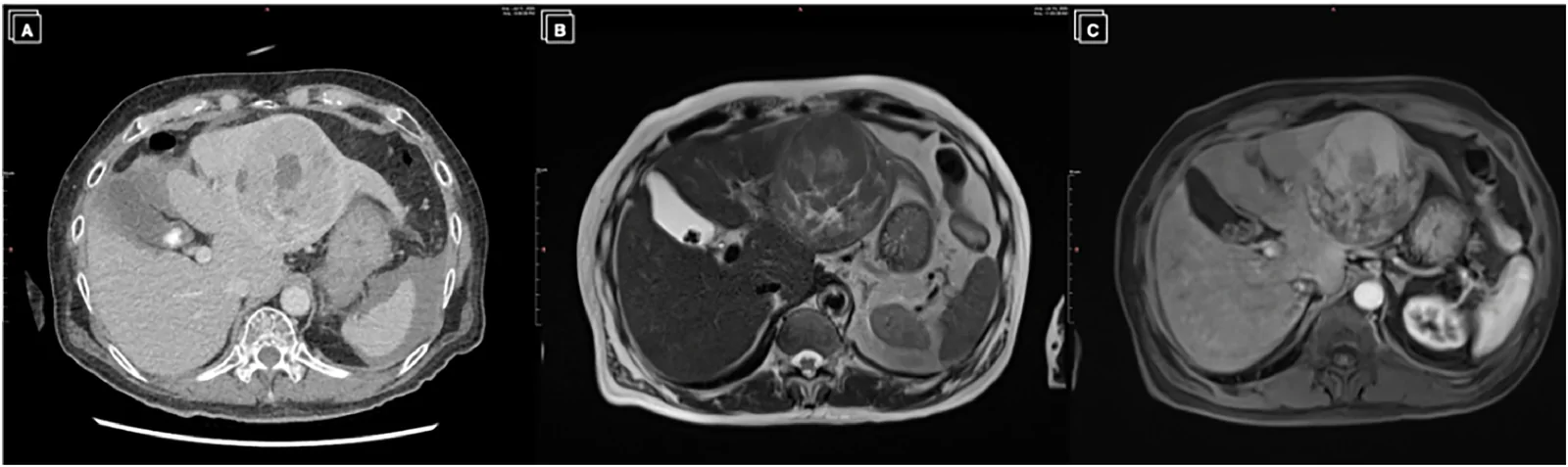
Pretreatment CT and MRI evaluation of a hemorrhagic hypervascular left hepatic lobe mass. (A) Contrast-enhanced CT venous phase obtained July 11 demonstrates a large heterogeneous mass centered in the left hepatic lobe with associated hemoperitoneum. (B) axial T2-weighted HASTE MRI obtained July 14 demonstrates internal heterogeneity within the mass, compatible with hemorrhagic and necrotic components. (C) Arterial-phase postcontrast T1-weighted VIBE MRI obtained July 14 demonstrates prominent hypervascular enhancement within the mass. Overall imaging findings raised concern for hemorrhagic hepatocellular carcinoma, later confirmed by biopsy.

Contrast-enhanced computed tomography of the abdomen performed on July 11, 2025, followed by magnetic resonance imaging on July 14, 2025, demonstrated a large, approximately 8-10 cm hypervascular mass within the left hepatic lobe, with intratumoral hemorrhage and associated hemoperitoneum (Fig. 1). These findings correlated with the patient’s acute blood-loss anemia and indicated an ongoing risk of recurrent hemorrhage.

General surgery documented that the approximately 10 cm hypervascular mass carried a substantial risk of rebleeding. Serial complete blood count monitoring, evaluation at a tertiary hepatobiliary center, and urgent interventional radiology involvement in the event of clinical instability were recommended. Because the patient lacked health insurance and his family initially declined transfer to a tertiary center, surgery recommended management by interventional radiology at the admitting institution.

Because the patient had no clearly established at-risk liver background and the imaging report did not document all major features required for formal Liver Imaging Reporting and Data System categorization, LI-RADS was not used to establish the diagnosis. Given the lesion’s large size, marked hypervascularity, intratumoral hemorrhage, hemoperitoneum, and risk of recurrent or catastrophic bleeding despite preserved hemodynamic stability, urgent embolization was pursued to achieve definitive arterial hemostasis. The available imaging reports did not describe morphologic features of cirrhosis, such as a nodular hepatic contour or surface irregularity.

On July 16, 2025, the patient underwent combined ultrasound-guided core biopsy and urgent transarterial particle embolization of the left hepatic artery [7,8].

**Fig. 2:**
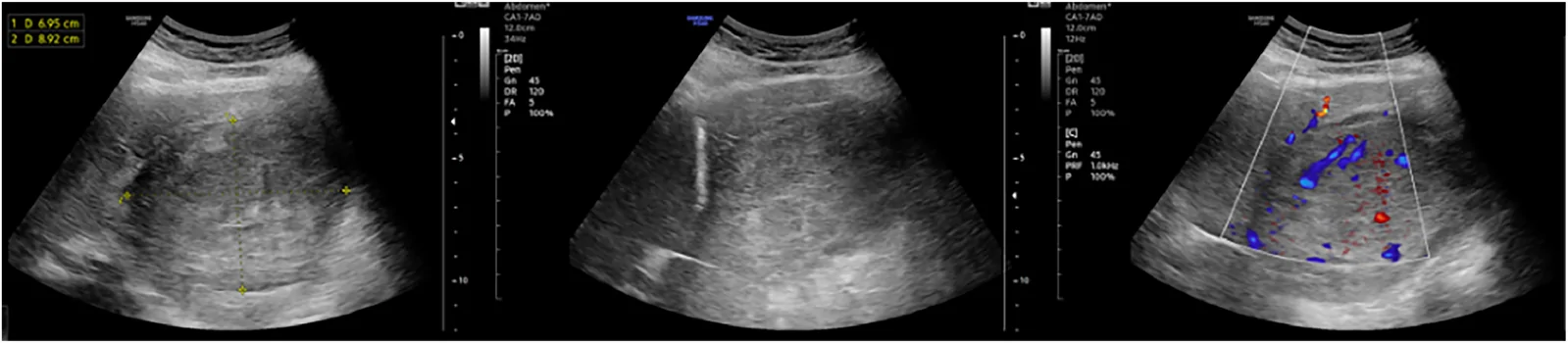
Ultrasound evaluation before biopsy and embolization. (A) B-mode ultrasound demonstrates a large heterogeneous left hepatic lobe mass with internal complexity compatible with hemorrhagic and necrotic components. (B) Ultrasound image obtained during biopsy planning demonstrates the selected needle trajectory through a relatively avascular portion of the lesion. (C) Color Doppler ultrasound demonstrates prominent internal vascularity, supporting the hypervascular nature of the mass. These findings guided selection of a safer biopsy trajectory while minimizing traversal of vascular tumor components.

Intra-procedural ultrasound demonstrated a large heterogeneous hepatic mass containing areas of intralesional hemorrhage and necrosis on B-mode imaging, as well as prominent internal vascularity on color Doppler imaging (Fig. 2). These findings confirmed the hypervascular nature of the lesion and allowed identification of a relatively avascular biopsy trajectory.

Under ultrasound guidance, a 17/18-gauge core biopsy device (Argon Medical Devices) was advanced into the left hepatic parenchyma, and 2 core biopsy specimens were obtained. The needle was subsequently redirected into the hepatic mass, and 3 additional core biopsy specimens were collected for histopathologic evaluation.

**Fig. 3:**
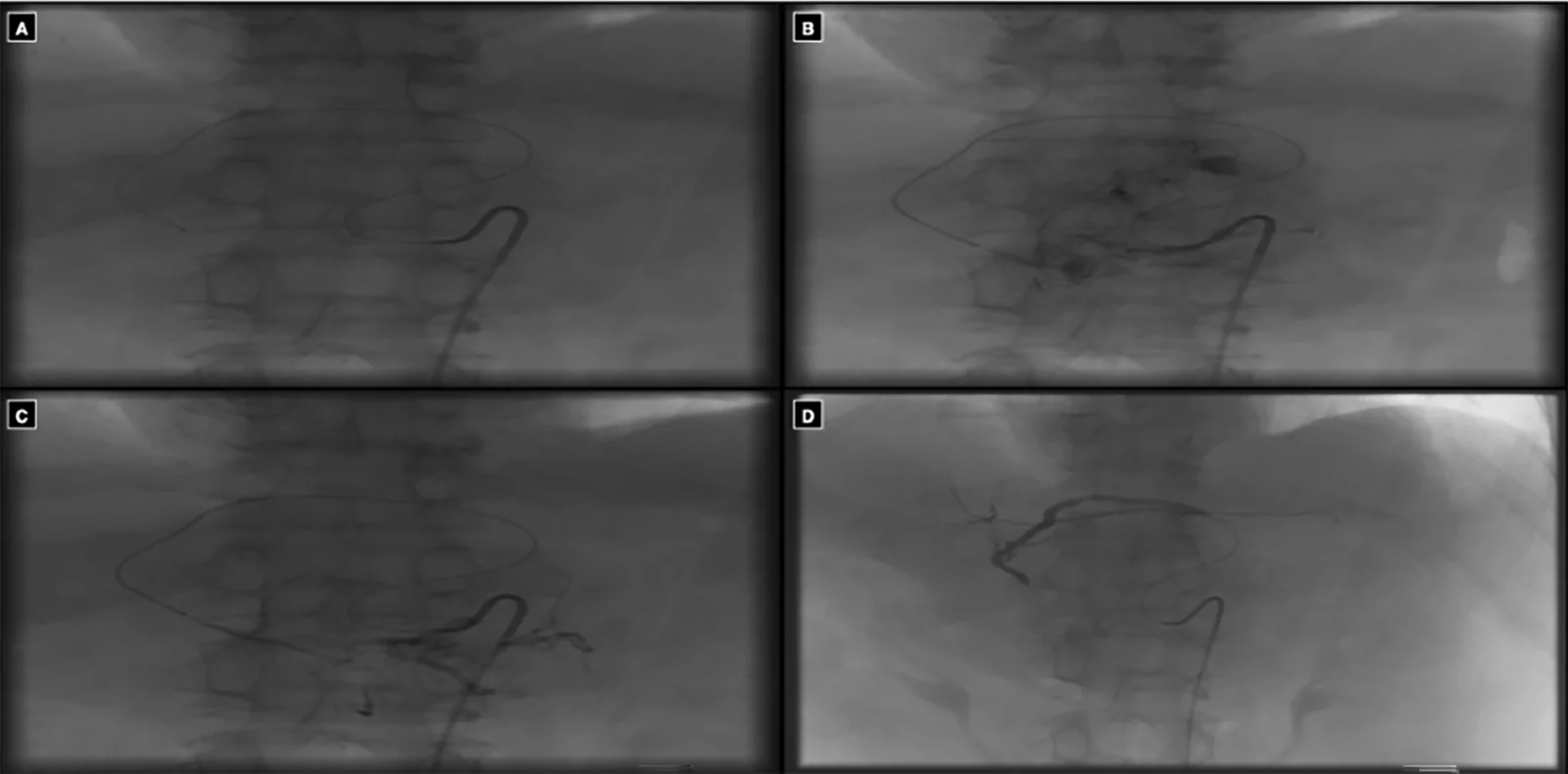
Transarterial particle embolization of the left hepatic artery. (A) selective left hepatic angiography demonstrates arterial supply to the hypervascular left hepatic lobe mass. (B) selective angiography demonstrates abnormal arterial tumor vascularity with irregular parenchymal blush and focal contrast pooling, corresponding to the known left hepatic lobe mass and concerning for a pseudoaneurysm or hemorrhagic vascular abnormality. (C) Postembolization angiography demonstrates marked reduction of abnormal tumor vascularity without obvious residual tumoral blush. (D) Completion angiography demonstrates cessation of arterial flow to the treated tumor territory without visible nontarget embolization.

Under moderate sedation and local anesthesia, vascular access was obtained through the right common femoral artery using a 6-French sheath. Celiac angiography demonstrated a left hepatic artery arising from a gastrohepatic trunk. Selective angiography revealed tumor supply from branches of the left hepatic artery, with focal contrast pooling and suspected pseudoaneurysm formation (Fig. 3). These angiographic findings further supported the risk of recurrent hemorrhage and the need for immediate arterial hemostasis.

A microcatheter was advanced into the feeding branch supplying the suspected pseudoaneurysm. Embolization was performed using 300-500 μm Embospheres (Merit Medical) until angiographic resolution of flow was achieved. Additional embolization of the remaining tumor supply was performed using 100-300 and 300-500 μm Embospheres.

Completion angiography demonstrated complete cessation of arterial flow to the tumor without evidence of nontarget embolization. Hemostasis at the vascular access site was achieved using a Mynx vascular closure device. No immediate procedural complications were observed. The patient was monitored overnight for postembolization syndrome and was subsequently discharged with follow-up arranged with interventional radiology and oncology.

**Fig. 4:**
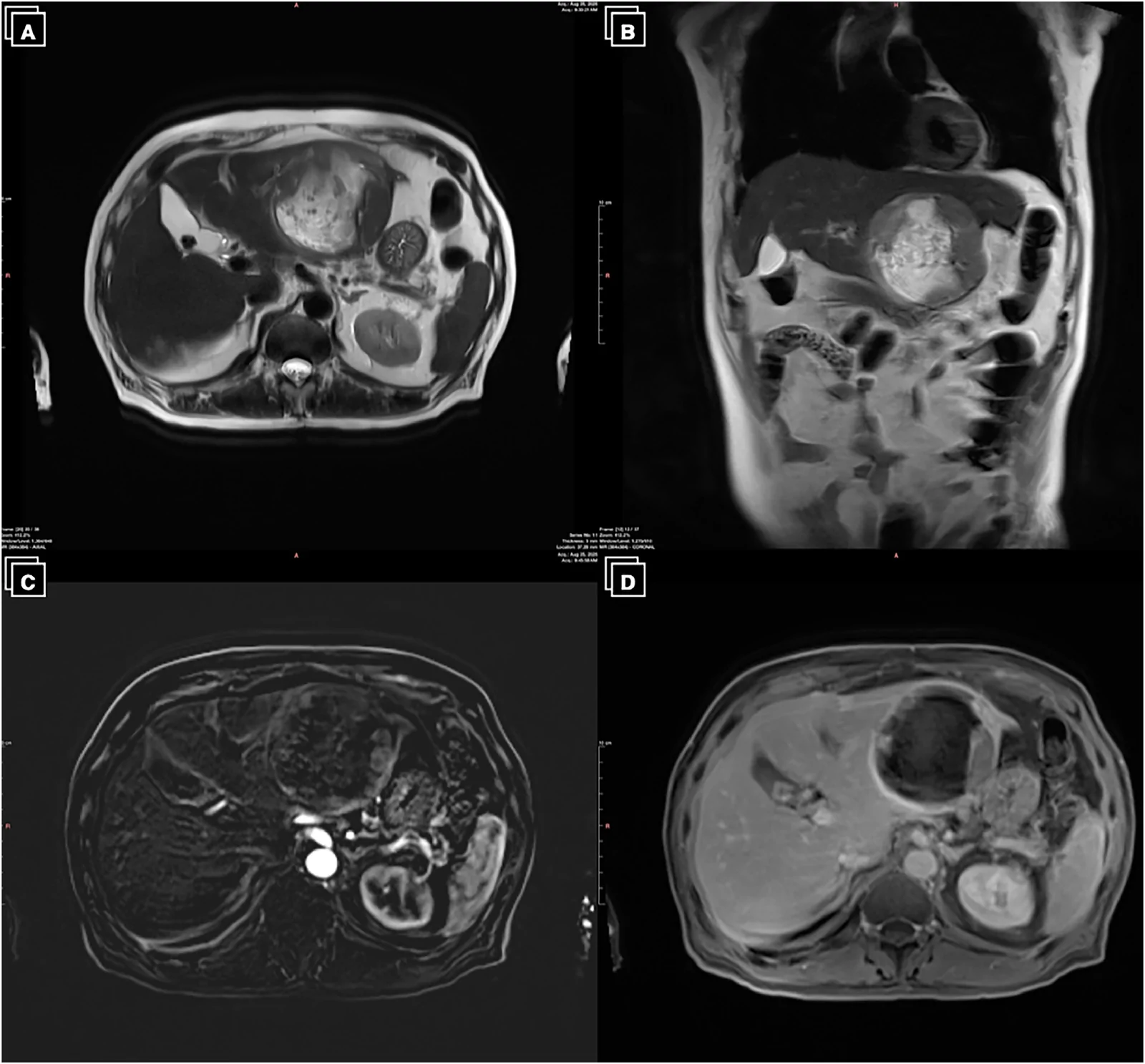
Follow-up MRI approximately 6 weeks after embolization. (A) Axial T2-weighted HASTE MRI obtained August 25 demonstrates a heterogeneous post-treatment cavity in the left hepatic lobe containing mixed signal material, compatible with necrotic debris and hemorrhagic or proteinaceous contents. (B) Coronal T2-weighted HASTE MRI further demonstrates the treated cavity and peripheral rim-like signal abnormality. (C) Axial early postcontrast subtraction image demonstrates no convincing central enhancement within the treated cavity, with peripheral rim-like enhancement along the treatment margin. (D) Delayed postcontrast MRI demonstrates persistent peripheral rim-like enhancement without convincing central enhancement.

Follow-up magnetic resonance imaging of the abdomen demonstrated expected postembolization changes within the treated left hepatic lobe lesion (Fig. 4). Axial and coronal T2-weighted half-Fourier acquisition single-shot turbo spin-echo images demonstrated a heterogeneous post-treatment cavity containing mixed-signal material compatible with necrotic debris and hemorrhagic or proteinaceous contents.

Early post-contrast subtraction and delayed postcontrast imaging demonstrated no convincing central enhancement, with persistent peripheral rim-like enhancement along the treatment margin. These findings were consistent with successful tumor devascularization and expected postprocedural evolution.

Histopathologic examination of the core biopsy specimens confirmed well- to moderately differentiated hepatocellular carcinoma, consistent with the imaging appearance of a hypervascular hepatic mass complicated by intratumoral hemorrhage. Detailed immunohistochemical staining results were not available in the records reviewed.

## Discussion

Spontaneous hemorrhage from hepatocellular carcinoma is an uncommon but potentially fatal presentation, occurring in approximately 3%-15% of HCC cases, with reported mortality rates ranging from 25% to 75% without prompt intervention [[3], [4], [5]]. The risk of spontaneous rupture increases with tumor size, with lesions greater than 5 cm carrying significantly higher risk [5,9]. In this case, the patient’s 8-10 cm left hepatic lobe mass demonstrated hemorrhagic and necrotic components with associated hemoperitoneum, presenting as severe acute blood loss anemia requiring urgent hemorrhage control.

### Clinical presentation and diagnostic challenges

The patient’s initial presentation with chest pain and new-onset seizure, rather than classic signs of hemoperitoneum such as abdominal pain and hemodynamic instability, highlights the varied and sometimes atypical presentations of hemorrhagic HCC [10,11]. The seizure was likely multifactorial, related to metabolic derangements and severe anemia from ongoing blood loss.

Although the patient did not develop overt hemorrhagic shock, he had severe anemia and a large hypervascular hepatic tumor with intratumoral hemorrhage and hemoperitoneum. Hemodynamic stability at a single point in time did not eliminate the substantial risk of recurrent or catastrophic bleeding.

General surgery specifically documented a high risk of rebleeding, and angiography subsequently demonstrated abnormal tumor vascularity with focal contrast pooling and suspected pseudoaneurysmal change. The decision to proceed with urgent embolization was therefore based on the need to achieve definitive arterial hemostasis before clinical decompensation.

Cross-sectional imaging demonstrated a large heterogeneous hypervascular hepatic mass with hemorrhagic and necrotic components and associated hemoperitoneum. Because no clearly established at-risk liver background was documented and imaging alone was not considered sufficiently specific to establish a definitive diagnosis, formal LI-RADS categorization was not relied upon and tissue confirmation was pursued.

Ultrasound with color Doppler demonstrated internal vascularity and guided selection of a relatively avascular biopsy trajectory. Pathologic evaluation confirmed well to moderately differentiated hepatocellular carcinoma.

### Role of transarterial embolization

Management of spontaneous HCC rupture has traditionally involved either emergency surgical resection or transarterial embolization [6,[12], [13], [14]]. Emergency hepatic resection, while potentially curative, carries substantial perioperative risk and is often not feasible in patients with poor liver reserve, significant comorbidities, or hemodynamic instability [12,15,16].

In contrast, transarterial embolization offers a minimally invasive alternative with reported success rates of 53%-100% for achieving hemostasis and lower procedural mortality [7,8,13].

Transarterial embolization works by occluding the arterial supply to the tumor, thereby controlling hemorrhage and inducing tumor necrosis through ischemia [[17], [18], [19], [20]]. The liver’s dual blood supply allows selective embolization of the hepatic artery feeding the tumor while preserving function of surrounding liver.

In this case, selective catheterization of the left hepatic artery with staged particle embolization using 100-300 and 300-500 μm microspheres achieved complete angiographic stasis without complications or evidence of nontarget embolization [[19], [20], [21]].

### Technical considerations and anatomic variants

Angiography demonstrated the left hepatic artery arising from a gastrohepatic trunk directly from the celiac axis. Recognition of such variants is critical for safe and effective embolization, as failure to identify aberrant vessels can result in incomplete treatment or inadvertent nontarget embolization [20,22].

Selective angiography allowed characterization of abnormal tumor vascularity, focal contrast pooling, and suspected pseudoaneurysmal change, which was targeted during embolization.

The decision to perform concurrent ultrasound-guided biopsy merits discussion. Although embolization could have been performed empirically because of the hemorrhagic hypervascular mass, tissue diagnosis was important for prognostic counseling, treatment planning, exclusion of alternative vascular tumors, and assessment of tumor differentiation.

Ultrasound guidance allowed identification of a relatively avascular trajectory, minimizing the risk of exacerbating hemorrhage during biopsy.

### Socioeconomic and access considerations

The patient’s lack of health insurance and his family’s initial reluctance to pursue transfer to a tertiary hepatobiliary center shaped the treatment course. Definitive hemorrhage control was therefore provided by the admitting institution’s interventional radiology service.

This case illustrates how interventional radiology can provide life-saving hemorrhage control and tumor devascularization when access to specialized hepatobiliary care is limited [2,23].

### Postembolization imaging and expected evolution

Follow-up MRI approximately 6 weeks after embolization demonstrated expected post-treatment evolution of the treated lesion. Imaging showed a central heterogeneous post-treatment cavity with mixed T2 signal, compatible with necrotic debris and hemorrhagic or proteinaceous contents following tumor devascularization.

Peripheral rim-like enhancement likely reflected treatment-margin change and reactive tissue. No imaging findings suggestive of abscess formation, such as intralesional gas or an air-fluid level, were identified. These findings were consistent with successful tumor devascularization [19,24,21].

### Patient outcome and limitations

The patient remained hemodynamically stable following embolization and was discharged home with outpatient follow-up arranged with interventional radiology and oncology. At discharge, discussions were ongoing regarding potential candidacy for definitive locoregional or systemic therapies, although financial constraints and limited access to specialized oncologic care remained significant barriers.

This case has several limitations. First, Viral hepatitis serologies and serum alpha-fetoprotein were not obtained during the index hospitalization because immediate clinical priority was placed on hemorrhage control and biopsy-based tissue diagnosis. These studies were deferred to outpatient oncologic evaluation.

Liver chemistry testing was available and demonstrated mild aminotransferase elevation, preserved bilirubin, and hypoalbuminemia. Although serum alpha-fetoprotein would have provided additional baseline tumor-marker information, it was not required to establish the diagnosis because histopathologic examination confirmed hepatocellular carcinoma.

Second, detailed immunohistochemical results were not available. Third, the patient’s long-term outcome and response to subsequent therapies are not yet known. Finally, transarterial embolization achieved acute hemostasis but was not intended as definitive curative therapy; ongoing multidisciplinary care and surveillance remain necessary [23].

## Conclusion

Urgent transarterial particle embolization is an effective and minimally invasive therapy for hemorrhagic hepatocellular carcinoma. In this case, preserved hemodynamics did not negate the need for intervention because the patient had severe anemia, a large hypervascular hemorrhagic tumor, focal contrast pooling with suspected pseudoaneurysmal change, and a documented high risk of rebleeding.

Early imaging recognition, multidisciplinary collaboration, and timely arterial hemostasis are essential to prevent catastrophic decompensation in this life-threatening presentation [3,7,8,13,15,16].

## Declaration of generative AI and AI-assisted technologies in the writing process

During the preparation of this manuscript, ChatGPT (OpenAI) was used to assist with language organization and clarity. The authors reviewed and edited all content and take full responsibility for the accuracy and integrity of the final manuscript.

## Patient consent

Written informed consent was obtained from the patient for publication of this case report and any accompanying images. The patient was informed that no identifying information would be disclosed, and a signed consent form is retained by the authors in accordance with institutional and journal policies.
